# What Is Menstruation?

**Published:** 1875-08

**Authors:** 


What is Menstruation?—Answers to this question have been numerous and varied. The last is that given by Dr. Jno. Williams, (*London Obstetrical Journal,* February–March, 1875.) His views are based upon the study of twelve uteri taken from women who had died from some general cause during different parts of the menstrual period. Microscopical drawings illustrate the paper.

1. He finds that the uterus is never at rest. It is ever undergoing those changes which either make it a fit receptacle for the ovum when impregnated, or which prepare it to carry off the ovum when impregnation has failed.

2. Preceding the menstrual flow a fatty change begins near the inner orifice and on the surface of the uterus, which finally covers the entire muscular wall.

3. It is quite probable that when completed this fatty degeneration induces contraction of the uterine muscular fibres.

4. This contraction of the muscular fibres forces the blood into the vessels of the mucous membrane.

5. This flow of blood into the mucous membrane expels the contents of the glands and the greater part of their lining epithelium. As the vessels have undergone fatty degeneration, they give way, and extravasation of blood into the mucous membrane takes place.

6. Rapid disintegration of this membrane now occurs, cell by cell, the process beginning immediately within the inner orifice, and proceeding along the surface towards the fundus, and at the same time towards the muscular wall. The removal of the membrane occupies from three to eight days. The walls of the vessels, as well as the surrounding tissue, being involved, openings are made in the vessels, and hemorrhage into the uterine cavity takes place.

7. While this destructive process is going on, a new membrane begins to form on the muscular wall, immediately subjacent to it. This begins near the inner orifice, and at the end of a week after the cessation of the catamenia, the whole body of the uterus is covered by mucous membrane. This membrane is produced by the proliferation of the elements of the muscular wall of the organ, the muscular fibres producing fusiform cells, the connective tissue; the round cells and the groups of round cells in the meshes formed by muscular bundles, the glandular epthelium.

8. At about the tenth day after the cessation of the catamenial flow, the entire membrane is covered with columna epithelium, and has reached the highest state of development in the unimpregnated uterus.

9. Failing to be impregnated, fatty degeneration of the membrane follows, and hemorrhage takes place, and in a few days the structure which had been formed during the preceding three weeks is carried off as sanguineous debris.—*Detroit Review.*

